# Psychological features of abstinent heroin users before and after rehabilitation in Saint Petersburg, Russia

**DOI:** 10.1186/s13104-018-3699-5

**Published:** 2018-08-14

**Authors:** Timofey Galankin, Dmitry Lioznov, Svetlana Nikolaenko, Louise-Anne McNutt, Emily Leckman-Westin, Perry F. Smith

**Affiliations:** 1 Laboratory of Pharmacoepidemiology and Pharmacokinetics, Pavlov First Saint Petersburg State Medical University, Lev Tolstoy Street 6/8, St. Petersburg, 197022 Russia; 2 Department of Infectious Diseases, Pavlov First Saint Petersburg State Medical University, Lev Tolstoy Street 6/8, St. Petersburg, 197022 Russia; 3 Department of Epidemiology and Biostatistics, School of Public Health, University at Albany, State University of New York, 1 University Place, Rensselaer, NY 12144 USA

**Keywords:** Opiates, Heroin, Addiction, Rehabilitation, MMPI, ASI

## Abstract

**Objective:**

The objective of the study was to describe psychological features of abstinent heroin users undergoing rehabilitation in Saint Petersburg, Russia. Study subjects (n = 197) were recruited prospectively at the time of their admission to rehabilitation between March 2010 and May 2011 at 7 inpatient opiate addiction rehabilitation centers in Saint-Petersburg and neighboring regions, Russia. The centers provided varying rehabilitation programs; 6 of them were religious centers. Socio-demographic information and self-reported HIV status were collected. Personality profiles and severity of drug-associated problems were estimated before and after rehabilitation using the Minnesota Multiphasic Personality Inventory 2 (MMPI-2), and the Addiction Severity Index (ASI).

**Results:**

Thirty-three (17%) subjects dropped out before completing rehabilitation (non-completers). All subjects (completers and non-completers) had psychopathological personality profiles according to MMPI-2. These profiles were refractory to clinically significant improvement after rehabilitation, although some statistically significant changes toward improvement were observed. ASI scores showed statistically and clinically significant improvements after rehabilitation on all scales. Participants in longer-term versus shorter-term rehabilitation programs showed similar changes in their pre- and post-rehabilitation MMPI-2 and ASI scores. Our results suggest that unmet psychiatric needs should be addressed to potentially improve treatment completion in this population.

**Electronic supplementary material:**

The online version of this article (10.1186/s13104-018-3699-5) contains supplementary material, which is available to authorized users.

## Introduction

Of the estimated 11–21 million people who inject drugs (PWID) in the world, many reside in China, the United States, the Russian Federation, and Brazil, which together account for an estimated 45% of the worldwide population of PWID [1, 2]. Drug treatment program designs should be informed by a solid understanding of the factors that predict better treatment outcomes. To date, few studies have evaluated drug treatment outcomes in Russia. This prospective study assessed the demographic and psychological characteristics of PWID at 7 inpatient opiate addiction rehabilitation centers in Russia in 2010–2011. The analysis of this study is published for the first time.

## Main text

### Methods

#### Participants and the rehabilitation centers

This study is the secondary analysis of the data collected during the project “implementation of a comprehensive program to overcome the HIV/AIDS epidemic with participation of religious organizations” conducted by the religious public organization “Diakonia” in Saint Petersburg, Russia, under support of United Nations Development Programme (UNDP/163/2008 by 01.10.2008).

Study participants were enrolled at the time of their admission into 7 inpatient opiate addiction rehabilitation centers in Saint-Petersburg and neighboring regions in Russia. Table 1 contains descriptive information about the centers and rehabilitation practices. All entrants were invited to participate in the study between March 2010 and May 2011.

**Table 1:** Basic characteristics of 7 rehabilitation centers

Organization #	1	2	3	4	5	6	7
Religious affiliation	Baptist	Russian orthodox	No	Protestant	Russian orthodox	Baptist	Russian orthodox
Funding	NGO	NGO	GO	NGO	NGO	NGO	NGO
Year of foundation	2004	2000	2000	2004	2000	2005	1998
N (males)	50	8	9	23	26	26	2
N (females)	37	1	12	NA	NA	3	NA
Length of rehabilitation	Short			Long			
In-patient rehab (days)	60	42	30	240	180	180	450
Post rehab stay^a^ (days)	180	90	NA	180	180	120	NA
HIV test at admission	No	Yes	Yes	Yes	No	Yes	Yes
Interaction with							
Detox centers	+	+	+	+	+	+	
Centers for AIDS		+	+	+	+	+	+
Infectious departments		+	+	+	+	+	
Approaches							
12 steps		+	+		+		
Churching	+	+		+	+	+	+
Movie-therapy	+	+	+	+	+	+	+
Art-therapy		+	+		+		
Psychotherapy		+		+	+	+	
Work with the family	+	+	+	+	+	+	+
Relapse program		+	+	+	+		+
Adaptation apartments	+	+			+	+	+

NA, not applicable; N, number of participants recruited in each center; GO, governmental funding; NGO, non-governmental; 1, Good Samaritan; 2, Megapolis-Medexpress; 3, City Narcological Clinic; 4, Night shelter ‘Mercy house’; 5, Diaconia; 6, Liberation; 7, St. George’s Parish

^a^Post rehab stay = in some centers patients were able to stay after the rehabilitation period was over. In this study participants were tested before and straight after the obligatory part of rehabilitation (“in-patient rehab”). + means “yes”

For acceptance into the rehabilitation centers, all clients had to meet the following requirements: have completed a detoxification program, be drug-free, be motivated to undergo rehabilitation, and meet gender requirements. Factors that excluded acceptance into or resulted in expulsion from the rehabilitation programs included lack of a firm will to discontinue drug use or breaking program rules (e.g., drug or alcohol consumption, sexual relations, aggressive behavior).

Prior to enlisting participants, this study was approved by the Ethics Committee of Pavlov Medical State University. All participants provided written, informed consent upon entering the study. There was no remuneration or other compensation for participation.

#### Collection of information

On entering rehabilitation, participants filled a battery of questionnaires: the Minnesota Multiphasic Personality Inventory-2 (MMPI-2), Addiction Severity Index (ASI), and a socio-demographic questionnaire. The subjects required about 60–90 min on average to fill the questionnaires, working individually with a psychologist (a member of the research team unaffiliated with the programs). At the end of rehabilitation, participants again filled the MMPI-2 and ASI surveys. Participants who failed to complete their rehabilitation programs were not retested.

The use of drugs or alcohol was prohibited at each center under the threat of expulsion from rehabilitation, if discovered. However, no tests were used to track subjects’ drug abstinence during rehabilitation.

The dates of early termination of rehabilitation by non-completers were not recorded by the centers, so there was no opportunity to analyze survival plots.

#### Questionnaires

Personality was assessed by the Russian version of the MMPI-2, which is a well-validated personality test for individuals aged 18 and older [3]. The MMPI-2 is a 567-item, true–false questionnaire that evaluates personality on 3 validity scales [lie (L), infrequency (F), and correction (K)], and 10 clinical scales [hypochondriasis (Hs), depression (D), hysteria (Hy), psychopathic deviate (Pd), masculinity–femininity (MF), paranoia (Pa), psychasthenia (Pt), schizophrenia (Sc), hypomania (Ma), and social introversion (Si)]. Standard T-scores are calculated for all 13 scales and are typically given the following general interpretations: scores higher than 65 reflect mild problems, higher than 75 suggest important psychological problems, and higher than 85 are typical of psychiatric inpatients [4]. The MMPI-2 is intended to assess an individual’s inherent character traits and usually provides very stable scores that do not change significantly over a 1 or even 5-year period [5].

The Addiction Severity Index-5 (ASI) is a well-documented instrument [6], administered as an interview with 164 items (some with multiple questions). It is intended to assess addiction severity during the prior 30-day period in 7 areas: medical, employment, drug use, alcohol use, family/social, legal, and psychiatric [7, 8]. The scores within each of the seven areas are reported on a decimal scale that range from 0 to 1, with higher scores indicating greater problem severity.

A socio-demographic form was developed specifically for this study. It contained 17 questions about socio-economic, infectious disease history and family status, and 9 questions about alcohol consumption.

#### Analysis

Calculations were performed using R 3.4.2 statistical package [9]. Categorical variables in the socio-demographic form were analyzed by the Fisher exact test; continuous variables were analyzed by the Wilcoxon rank-sum test. For analysis of the length of rehabilitation, duration of the rehabilitation programs was divided into short (2 months or less) and long (more than 2 months). The MMPI-2 scales were analyzed using multiple analysis of variance (MANOVA). Two MANOVA models were performed: between subjects MANOVA for completers vs. non-completers, and within subjects MANOVA for completers before/after rehabilitation. Post hoc pairwise comparisons were done with the t-tests: independent sample t-test was used to compare completers and non-completers, paired t-test was used to compare completers before and after rehabilitation. The ASI scales were analyzed by the Wilcoxon signed-rank test (before/after rehabilitation) and the Wilcoxon rank-sum test (completers/non-completers). Benjamini–Yekutieli (BY) method was used to control for erroneous rejections in multiple testing [10]. Missing values were excluded from the analysis.

To evaluate whether PWIDs in our sample could be classified into several meaningful subgroups, k-means cluster analysis of MMPI-2 or ASI scales was performed.

### Results

All rehabilitation clients who were invited to participate agreed to join the study. The study participants were 197 Caucasian adults (144 males, 53 females), ranging in age from 16 to 59 (Additional file 1: Table S1 and Additional file 2: Table S2). Of them, 80 (40.6%) were in long-term programs (6 months and longer), 117 (59.4%) were in short-term programs (1–2 months). Slightly more than half had completed secondary education, one-fourth were married or reported a partner, and about one-fourth reported being employed. Over half reported more than 9 years of addiction, and 59% reported undergoing previous detoxifications. Only 7% reported being homeless. There were no differences in socio-demographic characteristics between men and women, and between participants in shorter versus longer rehabilitation programs.

It was not possible to subdivide participants into meaningful subgroups by cluster analysis on the basis of their baseline MMPI-2 or ASI profiles. All participants bore very similar features on the MMPI-2 profile, forming one homogenous group.

**Fig. 1:**
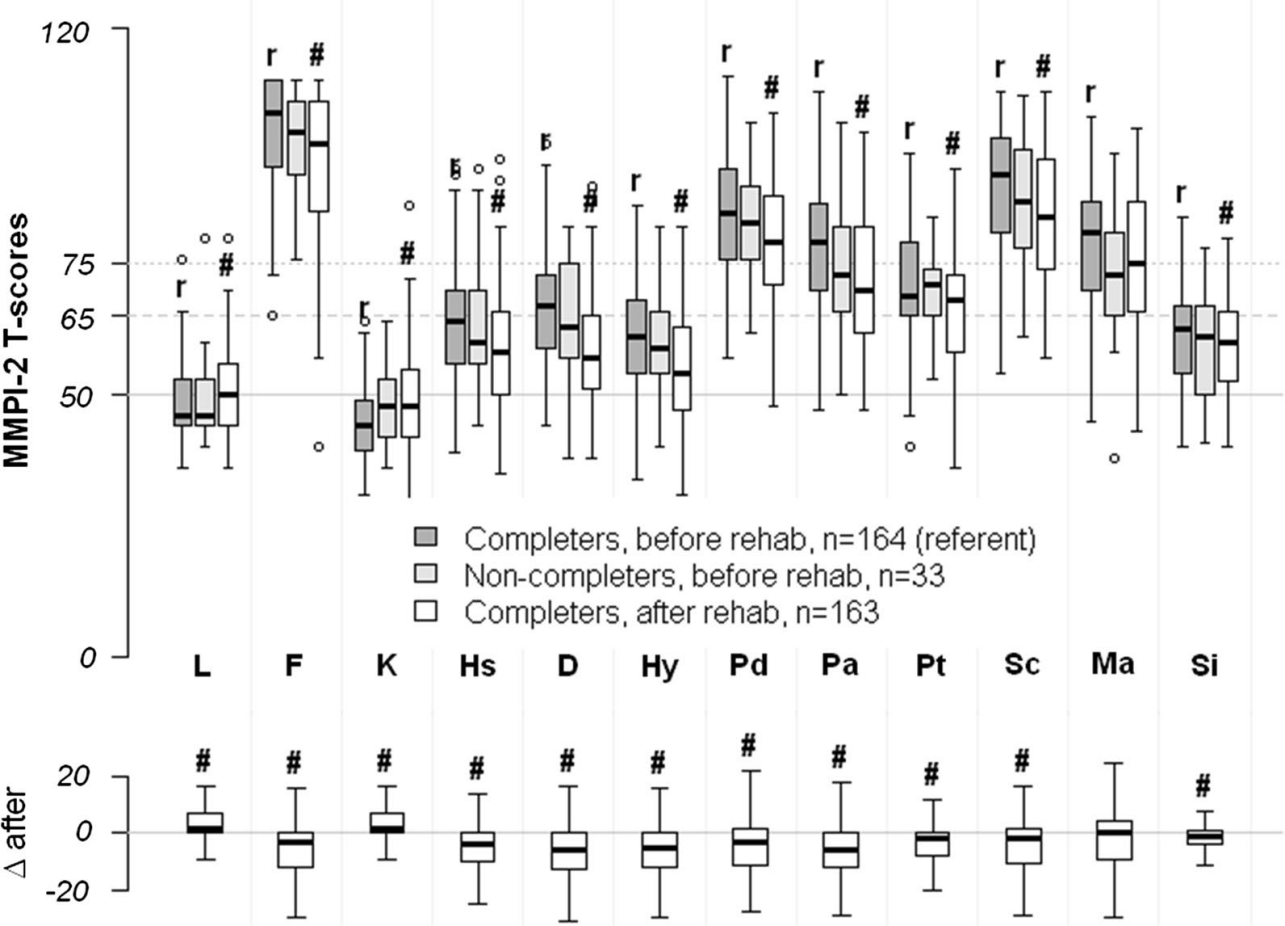
Changes in MMPI-2 scores after rehabilitation. Data are shown as MMPI-2 T-scores (upper boxplot), as well as difference in MMPI-2 T-scores after rehabilitation completion (lower boxplot). The lower boxplot demonstrates unidirectional changes of T-scores in the majority of completers after rehabilitation. The line at 50 is an absolute norm. The line at 65 depicts the border of normal values. The line at 75 depicts the border of severe disturbances. Statistical significance: ^#^p < 0.05 (BY-corrected for 24 two-sampled t-tests, paired for completers before/after rehabilitation, unpaired for completers before rehabilitation vs non-completers), r—referent group (completers before rehabilitation). MMPI-2 scales: L—lie, F—infrequency, K—correction, Hs—hypochondriasis, D—depression, Hy—hysteria, Pd—psychopathic deviate, Pa—paranoia, Pt—psychasthenia, Sc—schizophrenia, Ma—hypomania, Si—social introversion

**Fig. 2:**
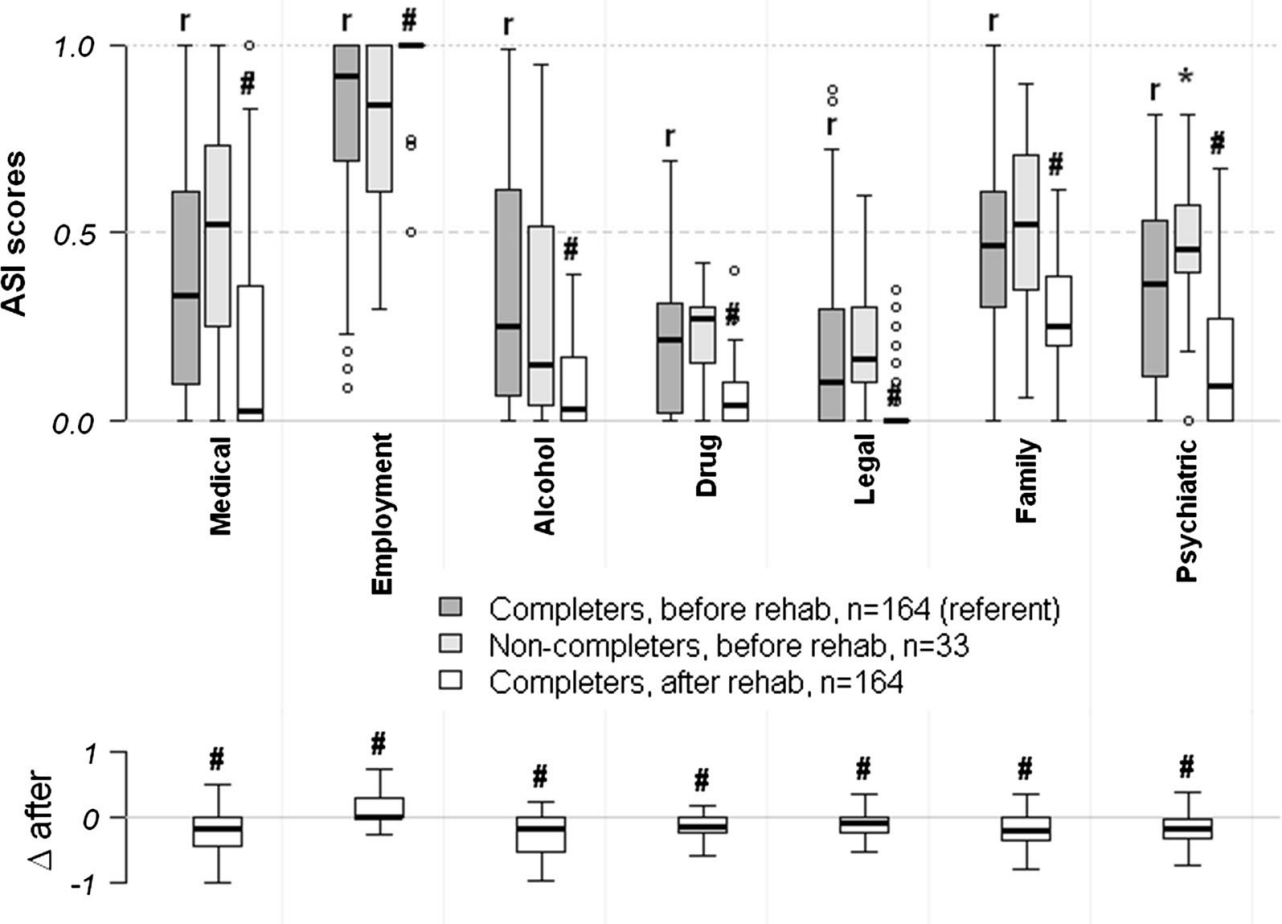
Changes in ASI and PES scores after rehabilitation. Data are shown as ASI scores (upper boxplot), as well as difference in ASI scores after rehabilitation completion (lower boxplot). The lower boxplot demonstrates unidirectional changes of ASI scores in the majority of completers after rehabilitation. The line at 0 is an absence of any addiction-associated problems, the line at 1 indicates severe problems. Statistical significance: *(non-completers, Wilcoxon rank-sum tests for independent samples), ^#^(completers, Wilcoxon signed-rank tests for paired samples) p < 0.05 (BY-corrected for all 14 two-sampled tests), r—referent group (completers before rehabilitation)

Of the 197 participants, 33 (17%) dropped out of their rehabilitation programs. These non-completers did not have any distinctions in socio-demographic variables (Additional file 1: Table S1 and Additional file 2: Table S2) or in MMPI-2 profile (MANOVA’s F = 0.93, p = 0.51, see also Fig. 1). The non-completers had slightly higher scores on ASI psychiatric scale (p < 0.05, Wilcoxon rank-sum test, BY correction, Fig. 2). The size of the dataset was insufficient to run a multivariate prediction model.

Also, the non-completion was associated with the length of rehabilitation: 24 (30.0%) of non-completers were in longer-term programs and only 9 (7.7%) in shorter-term programs (p = 0.0011, Fisher exact test). However the rate of non-completion (number of non-completers/total time of rehabilitation) was the same: 4.3 non-completers per 100 patients per month (30 days) in shorter-term programs and 4.4 non-completers per 100 patients per month in longer-term programs (p = 1.00, Poisson test).

Comparing pre- and post-rehabilitation scores, MMPI-2 results showed a subtle, but statistically significant, reduction in all clinical scales except for the Ma-scale, reduction in the F-scale, and an increase in the K and L scales (MANOVA’s F = 56.6, p < 0.0001, see also Fig. 1). Pre- and post-rehabilitation ASI scores showed statistically and clinically significant improvements on all scales when all centers were included (Fig. 2). Participants in longer-term versus shorter-term rehabilitation programs showed similar changes in their pre- and post-rehabilitation MMPI-2 and ASI scores (not shown).

### Discussion

It has been shown in previous publications that PWIDs can often be subdivided into several psychologically distinct subgroups representing psychologically distinct subpopulations who may differ in their pathogenesis of addiction, response to treatment and prognosis [11–14]. In our sample, all 197 participants demonstrated very similar MMPI-2 profiles, such that we were unable to subdivide them into meaningful clusters. Their personality profiles were very similar to what was reported by Krupitsky and colleagues [15] in another sample of PWID in Saint Petersburg, Russia. This may indicate the clinical severity of the clients presenting for rehabilitation in this region. For instance, the study participants demonstrated severely abnormal personality scores on psychological testing with a pronounced F (infrequency), Pd (psychopathic deviate), Pa (paranoia), Sc (schizophrenia), and Ma (hypomania) profile (Fig. 1), which is typical for patients with antisocial or borderline personality disorders [16, 17]. The MMPI-2 cannot be used to provide psychiatric diagnoses. Nevertheless, antisocial and borderline personality disorders are not an unusual finding in PWID [16, 18–22], and many studies have found similar psychopathic personality profiles in subgroups who use heroin and cocaine [11–14, 23, 24]. The main difference between our results and other studies is that other authors have usually found psychologically heterogeneous clusters [11–14]. Additionally, a majority of subjects in our study demonstrated extremely high scores on the F (infrequency) scale. In many studies, clients with MMPI-2 tests with F-scores higher than 90 (sometimes 100) are considered to be invalid and censored. We did not censor them in this study because the censoring rule should not be applied to patients for whom clinically important psychopathology cannot be ruled out [16].

We found that subjects’ MMPI-2 scores improved during rehabilitation, although the clinical significance of these changes is unclear. At the end of rehabilitation, participants demonstrated a subtle reduction in all clinical scales and the F-scale, and an increase in the K and L scales. The MMPI-2 profile is believed to be very stable in healthy volunteers and psychiatric patients, with no changes in average scores after several years [5]. These findings suggest that the MMPI-2 profile is a relatively stable characteristic in PWID as well.

We were unable to demonstrate any robust differences between the completers and non-completers, except that non-completers had higher levels of psychiatric problems (ASI) at the start of rehabilitation. The rate of dropping out seemed to be constant over time and was not associated with the length of rehabilitation.

We found no association between dropping out and variables such as family status, Family ASI scale, and having children. Other studies usually show a positive relationship between therapy retention and family involvement [25]. Also, we found no association with the length of addiction, probably because 91% of participants reported at least 3 years’ history of dependence, suggesting extremely severe drug abuse.

ASI scores showed statistically and clinically significant improvements on all scales after rehabilitation. These results were predictable. Since rehabilitation was in-patient, participants could not work or consume alcohol, which likely affected their Employment and alcohol ASI scales.

## Limitations

Our results should be interpreted with the following limitations in mind. The study participants had long-standing addiction and significantly abnormal psychiatric personality profiles that may not be typical of PWID in Russia, limiting the generalizability of these findings. Limited resources and patient confidentiality also prevented any laboratory, medical, or evaluations by psychiatric clinicians for our study, and no drug tests were performed during rehabilitation to assess adherence to abstinence. Additionally, we had no information on those dropping out of rehabilitation and no long-term follow-up data on relapse rates after completion of rehabilitation. Lastly, we reported statistical test results comparable to those reported in the literature. However, no randomization was conducted in this study, and thus p-values cannot provide probability inferences as they would in randomized trials.

## Additional files

**Additional file 1: Table S1.** Socio-demographic characteristics, continuous variables.

**Additional file 2: Table S2.** Socio-demographic characteristics, categorical variables.

## Abbreviations

AIDS: acquired immunodeficiency syndrome
ASI: Addiction Severity Index
BY: Benjamini–Yekutieli
D: depression scale (MMPI-2)
F: infrequency scale (MMPI-2)
HIV: human immunodeficiency virus
Hs: hypochondriasis scale (MMPI-2)
Hy: hysteria scale (MMPI-2)
K: correction scale (MMPI-2)
L: lie scale (MMPI-2)
Ma: hypomania scale (MMPI-2)
MANOVA: multiple analysis of variance
MF: masculinity–femininity scale (MMPI-2)
MMPI: Minnesota Multiphasic Personality Inventory
Pa: paranoia scale (MMPI-2)
Pd: psychopathic deviate scale (MMPI-2)
Pt: psychasthenia scale (MMPI-2)
PWID: people who inject drugs
Sc: schizophrenia scale (MMPI-2)
Si: social introversion scale (MMPI-2)
UNDP: United Nations Development Programme
