# Supplementary material for: Psychological features of abstinent heroin users before and after rehabilitation in Saint Petersburg, Russia
Source: BMC Res Notes. 2018 Aug 14;11:589. doi: 10.1186/s13104-018-3699-5 (PMC6092806; doi:10.1186/s13104-018-3699-5)
Supplement: Supplementary file 1 — Additional file 1: Table S1. Socio-demographic characteristics, continuous variables. [file 13104_2018_3699_MOESM1_ESM.docx]

Table S1. Socio-demographic characteristics, continuous variables.

| **Factor** | **Completers** | | | **Non-completers** | | | **p-value**^1^ | |
| --- | --- | --- | --- | --- | --- | --- | --- | --- |
|  | **N (%)** | **Mean** | **SD** | **N (%)** | **Mean** | **SD** | **No correction** | **BY correction** |
| Age | 164 (100.0) | 32.1 | 8.5 | 31 (100.0) | 31.2 | 6.5 | 0.81 | 1.00 |
| Beer, ml^2^ | 51 (31.1) | 1690 | 1105 | 6 (18.2) | 833 | 408 | 0.028 | 0.85 |
| Wine, ml^2^ | 39 (23.8) | 1214 | 1042 | 4 (12.1) | 563 | 377 | 0.17 | 1.00 |
| Vodka, ml^2^ | 58 (35.4) | 659 | 357 | 5 (15.2) | 290 | 125 | 0.0037 | 0.17 |
| Pure alcohol recalculation^2^ | 83 (50.6) | 315 | 245 | 7 (21.0) | 164 | 113 | 0.084 | 1.00 |

N = Number of respondents answering each question. ^1^ Wilcoxon rank-sum test, Benjamini-Yekutieli correction is applied for 24 multiple tests (Table S1 and Table S2). ^2^ Alcohol consumption was calculated for those who reported it (as average volume in milliliters of alcohol beverage that respondent drinks at once)
