# Supplementary material for: Psychological features of abstinent heroin users before and after rehabilitation in Saint Petersburg, Russia
Source: BMC Res Notes. 2018 Aug 14;11:589. doi: 10.1186/s13104-018-3699-5 (PMC6092806; doi:10.1186/s13104-018-3699-5)
Supplement: Supplementary file 2 — Additional file 2: Table S2. Socio-demographic characteristics, categorical variables. [file 13104_2018_3699_MOESM2_ESM.docx]

Table S2. Socio-demographic characteristics, categorical variables.

| **Factor** | **Completers** | **Non-completers** | **p-value** | |
| --- | --- | --- | --- | --- |
|  | **N (%)** | **N (%)** | **No correction** | **BY correction** |
| **Gender** | | | | |
| Male | 120 (73.2) | 23 (69.7) | 1.00 | 1.00 |
| Female | 44 (26.8) | 9 (27.3) |  |  |
| Missing | 0 (0.0) | 1 (3.0) | -- | -- |
| **Education** | | | | |
| Secondary (≤10 years) | 64 (39.0) | 14 (42.4) | 0.84 | 1.00 |
| Specialized (> 10 years) | 84 (51.2) | 16 (48.5) |  |  |
| University level | 16 (9.8) | 2 (6.1) |  |  |
| Missing | 0 (0.0) | 1 (3.0) | -- |  |
| **Employment** | | | | |
| Yes | 46 (28.0) | 9 (27.3) | 1.00 | 1.00 |
| No | 117 (71.3) | 23 (69.7) |  |  |
| Missing | 1 (0.6) | 1 (3.0) | -- | -- |
| **Income per month** | | | | |
| No income | 16 (9.8) | 3 (9.1) | 0.059 | 1.00 |
| < $300 | 65 (39.6) | 7 (21.2) |  |  |
| > $300 | 66 (40.2) | 15 (45.5) |  |  |
| > $600 | 13 (7.9) | 7 (21.2) |  |  |
| Missing | 4 (2.4) | 1 (3.0) | -- | -- |
| **Family status** | | | | |
| Single | 83 (50.6) | 22 (66.7) | 0.21 | 1.00 |
| Marriage/Partner | 44 (26.8) | 5 (15.2) |  |  |
| Divorced/Widowed | 37 (22.6) | 5 (15.2) |  |  |
| Missing | 0 (0.0) | 1 (3.0) | -- | -- |
| **Have children** | | | | |
| Yes | 70 (42.7) | 8 (24.2) | 0.076 | 1.00 |
| No | 94 (57.3) | 24 (72.7) |  |  |
| Missing | 0 (0.0) | 1 (3.0) | -- | -- |
| **History of TB** | | | | |
| Yes | 5 (3.0) | 2 (6.1) | 0.27 | 1.00 |
| No | 138 (84.1) | 22 (66.7) |  |  |
| Missing | 21 (12.8) | 9 (27.3) | -- | -- |
| **HBV** | | | | |
| Yes | 30 (18.3) | 8 (24.2) | 0.19 | 1.00 |
| No | 113 (68.9( | 16 (48.5) |  |  |
| Missing | 21 (12.8) | 9 (27.3) | -- | -- |
| **HCV** | | | | |
| Yes | 102 (62.2) | 24 (72.7) | **0.0013** | **0.12** |
| No | 41 (25.0) | 0 (0.0) |  |  |
| Missing | 21 (12.8) | 9 (27.3) | -- | -- |
| **History of syphilis/ Ghonorrhea** | | | | |
| Yes | 22 (13.4) | 3 (9.1) | 1.00 | 1.00 |
| No | 121 (73.8) | 21 (63.6) |  |  |
| Missing | 21 (12.8) | 9 (27.3) | -- | -- |
| **HIV status** | | | | |
| Positive | 30 (18.3) | 10 (30.3) | 0.065 | 1.00 |
| Negative | 85 (51.8) | 16 (48.5) |  |  |
| Unknown | 42 (25.6) | 3 (9.1) |  |  |
| Missing | 7 (4.3) | 4 (12.1) | -- | -- |
| **IV route of HIV infection** | | | | |
| Yes | 25 (83.3) | 10 (100.0) | 0.56 | 1.00 |
| No | 4 (13.3) | 0 (0.0) |  |  |
| Missing | 1 (3.3) | 0 | -- | -- |
| **Length of addiction** | | | | |
| < 1 year | 5 (3.0) | 0 (0.0) | 0.74 | 1.00 |
| 1-3 years | 8 (4.9) | 1 (3.0) |  |  |
| 3-6 years | 19 (11.6) | 6 (18.2) |  |  |
| 6-9 years | 22 (13.4) | 5 (15.2) |  |  |
| > 9 years | 76 (46.3) | 13 (39.4) |  |  |
| Missing | 34 (20.7) | 8 (24.2) | -- | -- |
| **Detox history** | | | | |
| Yes | 92 (56.1) | 18 (54.5) | 0.84 | 1.00 |
| No | 66 (40.2) | 11 (33.3) |  |  |
| Missing | 6 (3.7) | 4 (12.1) | -- | -- |
| **History of rehabilitation** | | | | |
| Yes | 69 (42.1) | 17 (51.5) | 0.23 | 1.00 |
| No | 91 (55.5) | 13 (39.4) |  |  |
| Missing | 4 (2.4) | 3 (9.1) | -- | -- |
| **Heroine abstinence** | | | | |
| < 6 months | 46 (28.0) | 5 (15.2) | 0.20 | 1.00 |
| 6 - 23 months | 47 (28.7) | 10 (30.3) |  |  |
| ≥ 24 months | 44 (26.8) | 13 (39.4) |  |  |
| Missing | 27 (16.5) | 5 (15.2) | -- | -- |
| **Alcohol (self-reported)** | | | | |
| Sober | 69 (42.1) | 19 (57.6) | 0.12 | 1.00 |
| Once in a month or rarer | 50 (30.5) | 4 (12.1) |  |  |
| 2-3 times per month | 29 (17.7) | 4 (12.1) |  |  |
| 2-3 times per week | 10 (6.1) | 0 (0.0) |  |  |
| every day | 3 (1.8) | 0 (0.0) |  |  |
| Missing | 3 (1.8) | 6 (18.2) | -- | -- |
| **History of imprisonment** | | | | |
| Yes | 46 (28.0) | 12 (36.4) | 0.50 | 1.00 |
| No | 118 (72.0) | 20 (60.6) |  |  |
| Missing | 0 (0.0) | 1 (3.0) | -- | -- |
| **Homeless** | | | | |
| Yes | 11 (6.7) | 3 (9.1) | 0.71 | 1.00 |
| No | 152 (92.7) | 29 (87.9) |  |  |
| Missing | 1 (0.6) | 1 (3.0) | -- | -- |

N = Number of respondents answering each question. ^1^ Fisher exact test, Benjamini-Yekutieli correction is applied for 24 multiple tests (Table S1 and Table S2).
